# Combined use of Bumetanide and MGE cell transplantation alleviates neuropathic pain and its mechanism after spinal cord injury in mice

**DOI:** 10.3389/fimmu.2026.1751436

**Published:** 2026-03-18

**Authors:** Yang Yu, Fangyong Wang

**Affiliations:** 1 School of Rehabilitation, Capital Medical University, Beijing, China; 2 Department of Spine Surgery, Beijing Bo’ai Hospital, China Rehabilitation Research Center, Beijing, China; 3 School of Biological Science and Medical Engineering, Beihang University, Beijing, China; 4 University of Health and Rehabilitation Sciences, Qingdao, Shandong, China; 5 The First Clinical College, Shandong University, Jinan, Shandong, China; 6 School of Rehabilitation Medicine, Shandong University of Traditional Chinese Medicine, Jinan, Shandong, China

**Keywords:** GABAergic, MGE, neuroinflammation, neuropathic pain, NF-κB pathway, NKCC1, spinal cord injury

## Abstract

**Introduction:**

Neuropathic pain (NP) after spinal cord injury (SCI) is intractable with limited efficacy of single treatments. This study investigated the additive analgesic effect and molecular mechanisms of Bumetanide (Bu, a NKCC1 inhibitor) combined with medial ganglionic eminence (MGE) cell transplantation on SCI-induced NP.

**Methods:**

Ninety adult female C57BL/6N mice were randomly divided into 5 groups (Sham, SCI, Bu, Mge, Bu+Mge). A T10 moderate spinal cord contusion model was established, with treatments (Bu intraperitoneal injection and MGE orthotopic transplantation) on day 10 post-surgery. Behavioral assessments, ELISA, Western blotting, qRT-PCR, and immunofluorescence staining were used.

**Results:**

Compared with monotherapy, Bu+Mge significantly relieved SCI-induced NP. Mechanistically, it alleviated inflammation by inhibiting NF-κB pathway and microglia activation, rectified spinal cord and dorsal root ganglion NKCC1/KCC2 imbalance, increased GABA-A receptors and GAD65/67 mRNA, reduced glial scarring, and protected neurons, axons and myelin sheaths.

**Discussion:**

Bu combined with MGE cell transplantation relieves SCI-induced NP via multiple additive mechanisms, providing a novel theoretical basis and potential clinical strategy for NP treatment.

## 1 Introduction

As a refractory complication following spinal cord injury (SCI), neuropathic pain (NP) has become a major global health challenge afflicting millions of patients (1). Its incidence rate is as high as 70% (2), among which more than 40% of patients suffer from moderate to severe pain, with the disease course often lasting for several years or even a lifetime. The typical characteristics of the NP include spontaneous burning pain, needle-like pain, hyperalgesia (severe pain in response to mild stimuli) and allodynia (pain triggered by non-painful stimuli) (3). Pain not only leads to psychological problems such as sleep disorders, anxiety, and depression in patients but also severely restricts their physical activity and social participation, significantly impairing their quality of life.

Notably, existing treatment modalities have obvious limitations: First-line drugs such as Pregabalin and Gabapentin are effective in only 30%-40% of patients, and long-term use is associated with side effects, including somnolence and cognitive impairment (4). Although opioid drugs exhibit significant short-term analgesic effects, issues of addiction and tolerance restrict their clinical application (5). Surgical interventions such as spinal cord stimulation (SCS) are difficult to widely promote in clinical practice because of their high invasiveness, high cost, and significant individual differences in efficacy (6). Intrathecal injection can bypass the blood-spinal cord barrier to improve local bioavailability, yet it has extremely high operational accuracy requirements. It is prone to causing complications such as secondary SCI, dural fibrosis, or puncture site infection, which further impede nerve repair (7). NP following SCI is a key factor that affects patients’ activities of daily living and reduces their quality of life, but current clinical treatment methods have suboptimal efficacy. Therefore, exploring novel safe and effective therapeutic strategies to provide new ideas for the clinical treatment and rehabilitation of SCI-NP has become an important scientific issue that urgently needs to be addressed in the field of SCI.

From the perspective of pathophysiological mechanisms, it is generally accepted that the development of SCI-NP is the result of the combined action of multiple factors, and the dysregulation of inhibitory neural circuits is among the core mechanisms involved (8). Under normal physiological conditions, γ-aminobutyric acid (GABA)-ergic interneurons release GABA neurotransmitters to mediate postsynaptic chloride ion (Cl^-^) influx and induce hyperpolarization, thereby inhibiting the transmission of pain signals to the central nervous system. However, following SCI, massive release of excitatory neurotransmitters (such as glutamate) occurs in the injured area, which activates N-methyl-D-aspartate (NMDA) receptors and triggers excessive neuronal excitation (9). Moreover, the impairment of the Cl^-^ homeostasis regulatory mechanism leads to the loss of GABAergic inhibitory effects, which in turn increases neuronal excitability (10).

Sodium-potassium-chloride cotransporter 1 (NKCC1) (11) is a membrane protein that regulates chloride ion homeostasis in neurons and glial cells, and its expression and activation status determine the function of GABAergic receptors. SCI can result in upregulated expression of NKCC1, which actively transports chloride ions into neurons, thereby causing GABA to exert a depolarizing effect on the postsynaptic membrane. This effect not only deprives neurons of their inhibitory function but also increases the transmission of pain signals. Additionally, NKCC1 dysfunction can activate microglia and astrocytes, promoting the release of inflammatory factors such as tumor necrosis factor-α (TNF-α) and interleukin-1β (IL-1β), which further exacerbate neuroinflammatory responses and pain sensitization (12).

In recent years, the exploration of the therapeutic potential of NKCC1 inhibitors has attracted widespread attention. As a highly potent and specific NKCC1 inhibitor, the efficacy of Bumetanide (Bu) has been validated in multiple animal experiments. A study by Yan et al. (13) revealed that Bu can alleviate ionic homeostasis imbalance, cytotoxic edema, and neuronal death induced by acute SCI, thereby reducing spinal cord edema and minimizing spinal cord tissue damage. Another study (14) demonstrated that Bu can downregulate the expression level of NKCC1 in the dorsal horn of the spinal cord to regulate the intracellular Cl^-^ concentration. This process alters the function of GABAergic receptors and activates presynaptic inhibition, significantly alleviating abnormal mechanical pain and thermal hyperalgesia in rats after SCI. However, NKCC1 inhibitors have notable limitations (15): they can correct only Cl^-^ homeostasis in the short term and cannot improve the structural damage to neural circuits in the injured area. Moreover, long-term use is likely to impair renal chloride reabsorption, leading to electrolyte disturbances. Therefore, monotherapy with NKCC1 inhibitors is insufficient to achieve a long-term and stable analgesic effect.

With breakthrough advancements in stem cell technology and the field of neural repair, cell transplantation strategies have provided a new approach for the reconstruction of damaged neural circuits. The medial ganglionic eminence (MGE) originates from the ventral forebrain region during the embryonic stage. As a type of neural progenitor cell, it can migrate long distances, survive within the brain, and differentiate into GABAergic interneurons, participating in the formation of neural circuits and enhancing the function of the GABAergic nervous system (16). Bráz et al. (17) demonstrated that MGE cells can survive and differentiate into mature neurons in the spinal cord and exhibit therapeutic potential in various neuropathic pain models. In another study (18) were transplanted MGE cells into the spinal cords of adult mice. Immunofluorescence tracing confirmed that MGE cells can establish synaptic connections with projection neurons in the host spinal cord gray matter. By differentiating into inhibitory interneurons, MGE cells inhibit excessive spinal cord excitation, thereby alleviating mechanical hypersensitivity and neuralgia caused by peripheral nerve injury. Using electrophysiology and immunohistochemistry techniques, Etlin A et al. (19) reported that MGE cells can form functional GABAergic synaptic connections with spinal cord tissue in a spared nerve injury model, which further clarified the integration mechanism between transplanted MGE cells and host spinal cord neural circuits. Fandel TM et al. (20) transplanted human embryonic stem cell-derived MGE cells into the lumbar spinal cord of mice with thoracic SCI and reported that the transplanted cells could migrate to the injury site, develop into mature neurons, and establish synaptic connections with local spinal cord circuits, ultimately improving bladder function and alleviating pain. This study provided important experimental evidence for the application of MGE cell therapy in repairing damaged spinal cord tissue.

In summary, Bu can rapidly regulate Cl^-^ homeostasis to alleviate pain symptoms during the acute pain phase while creating a favorable microenvironment for the survival and differentiation of transplanted cells. In contrast, MGE cell transplantation can compensate for the limitation of the drug’s short-term effect by long-term reconstruction of functional inhibitory neural circuits and lead to synaptic connections and functional integration with the host spinal cord. The additive effect of these two interventions may cover different phases and pathological links in pain development: it can not only correct ionic homeostasis abnormalities but also repair the structure of neural circuits, while inhibiting neuroinflammatory responses. Therefore, this study aimed to establish a mouse model of SCI-NP, use Bu in combination with MGE cell transplantation as an intervention, systematically investigate its additive analgesic effect and potential molecular mechanisms, and provide an experimental basis and theoretical support for the combination therapeutic strategy for SCI-NP.

## 2 Methods

### 2.1 Animals

In this study, 5 adult female C57BL/6N mice (gestational days 12.5–14.5, specific pathogen-free (SPF) grade) and 90 adult female C57BL/6N mice (10 weeks old, body weight 16–20 g, SPF grade) were used. Female mice were selected based on previous evidence: a meta-analysis (21) revealed they account for 73.4% of subjects in SCI-induced NP models and exhibit stronger pain sensitivity, more stable NP phenotypes, and lower inter-individual variability than males (22), reducing experimental noise; manual bladder emptying after SCI is easier to perform (23), lowering urinary tract infection incidence; and their docile trait facilitates smooth experimental interventions and post-procedural care. And the sample size was determined by the resource equation method (24, 25), with considerations for the study design involving multiple endpoint time points and various experimental assays. All mice were provided by Speifu Biotechnology Co., Ltd. (Beijing, China; license no.: SCXK (Jing) 2024-0001). The mice were housed in a quiet environment with a 12-hour light/dark cycle at a temperature of 22 ± 2 °C and humidity of 55% ± 10%, with free access to food and water. This study was approved by the Institutional Animal Care and Use Committee of Capital Medical University (approval no.: AEEI-2025-233). Surgical procedures and post-operative animal care were performed in strict accordance with the guidelines of the Committee for the Purpose of Control and Supervision of Experiments on Animals. All operations aimed to minimize the number of experimental animals and their suffering.

### 2.2 Extraction and identification of MGE cells

#### 2.2.1 MGE extraction

Pregnant mice were anesthetized via intraperitoneal injection of tribromoethanol (Avertin: Dowobio, Shanghai, China; 20 ml/kg). After disinfection with 75% ethanol, the skin was longitudinally incised along the abdominal midline to expose the abdominal organs and uterus. At this point, the pregnant mouse was still under deep anesthesia. An immediate cervical dislocation operation was performed to euthanize it, ensuring its swift and painless death. The uterus and intrauterine fetal mice were completely removed and transferred to Petri dishes containing phosphate-buffered saline (PBS) supplemented with 1× penicillin−streptomycin. Under a stereomicroscope, the fetal mice were removed, and the scalp, skull, and meninges of the fetal mouse embryos were quickly peeled off to expose the intact brain tissue. After the cerebral cortex was cut, the lateral ventricles of the brain were observed to have a higher translucency than the other tissues; a raised area with the lowest translucency, presenting a “Y”-shaped protrusion, was located on the ventrolateral wall of the lateral ventricles—this area represented the ganglionic eminence (26). The upper part of the “Y” arms was the lateral ganglionic eminence, and the lower part was the MGE (**Supplementary Figure 1**). The bilateral MGE brain tissues were carefully and meticulously separated and placed into prechilled EP tubes, and the entire process was performed on ice. The MGE tissues were removed with sterile forceps and placed into 2 ml of prechilled (4 °C) 0.25% trypsin solution. They were minced into small pieces of approximately 1 mm³ using ophthalmic scissors and then pipetted with a 1 ml pipette to disperse the tissue pieces. The mixture was placed in a cell incubator for digestion for 10 minutes. Afterwards, 3 ml of seeding medium was added to neutralize the trypsin, and all the cell filtrates were collected into centrifuge tubes. The tissue debris was filtered out using a 200-mesh sterile filter, and the centrifuge tubes were centrifuged at 9000×g for 5 minutes. The cell pellet was retained, the supernatant was discarded, and fresh, rewarmed serum-free complete medium was added to resuspend the cells. The cells were cultured in an incubator at 37 °C, 5% CO_2_, and saturated humidity. An appropriate amount of fresh medium was added every 2 days, and the morphological changes of the cells were observed under an inverted phase-contrast microscope during the culture period.

#### 2.2.2 MGE identification

On days 6 and 11 of cell culture, immunofluorescence (IF) staining was used to observe the colocalization of the specific marker of MGE precursor cells, thyroid transcription factor 1 (Nkx2.1) and the GABA inhibitory interneuron marker glutamate decarboxylase 67 kDa (GAD67) under a laser confocal microscope (Nikon ECLIPSE TI-S, Japan) at 100× and 400× magnification. For detailed antibody information, see **Table 1**; for the detailed IF protocol, refer to Section 2.11.

**Table 1:** Antibody information.

Antibody	Host	Dilution (WB/IF)	Catalog number	Supplier	Application
IκB	Mouse	1: 1000/ 1: 400	4814T	Cell Signaling	WB/IF
P65	Rabbit	1: 1000/ 1: 400	8242T	Cell Signaling	WB/IF
TNF-α	Rabbit	1: 1000/ 1: 500	ab307164	Abcam	WB/IF
NKCC1	Rabbit	1: 1000/ 1: 100	ab303518	Abcam	WB/IF
KCC2	Rabbit	1: 1000/ 1: 100	94725T	Cell Signaling	WB/IF
GABA-A	Rabbit	1: 1000/ 1: 100	ab307359	Abcam	WB/IF
β-actin	Rabbit	1: 3000	AF7018	Affinity	WB
H3	Rabbit	1: 1000	Ab176842	Abcam	WB
HRP-Goat anti-Mouse IgG	Goat	1: 5000	GB23301	Servicebio	WB
HRP-Goat anti-Rabbit IgG	Goat	1: 5000	ZB-2301	ZSGB-BIO	WB
Iba-1	Rabbit	1: 200	YM8165	Immunoway	IF
GFAP	Rabbit	1: 200	16825-1-AP	Proteintech	IF
MBP	Mouse	1: 200	YM4830	Immunoway	IF
Tuj1	Rabbit	1: 200	YM8365	Immunoway	IF
NF200	Rabbit	1:200	18934-1-AP	Proteintech	IF
Nkx2.1	Rabbit	1:25	ab227652	Abcam	IF
GAD67	Mouse	1:200	67648-1-Ig	Proteintech	IF
VGAT	Rabbit	1: 100	Ab308062	Abcam	IF
Goat pAb to Rb lgG	Goat	1:500	Ab150077	Abcam	IF

WB, Western blot; IF, immunofluorescence; IκB, Inhibitor of κB; P65, Nuclear factor κB p65 subunit; TNF-α, tumor necrosis factor-α; NKCC1, Sodium-potassium-chloride cotransporter 1; KCC2, potassium-chloride cotransporter 2; GABA-A, γ-Aminobutyric acid type A; H3, Histone H3; Iba-1, ionized calcium-binding adapter molecule 1; GFAP, glial fibrillary acidic protein; MBP, myelin basic protein; Tuj1, β-Tubulin III; NF200, neurofilament 200 kDa protein; Nkx2-1:thyroid transcription factor 1; GAD67, glutamate decarboxylase 67 kDa; VGAT, Vesicular GABA Transporter.

### 2.3 SCI model

The mice were anesthetized with isoflurane throughout the surgical procedure (induction concentration of 5%, maintenance concentration of 2%). Hair within an area of approximately 1×2 cm was clipped, and after disinfection with 75% ethanol, a 1–2 cm incision was made at the T10 spinal segment. The skin and fascia were incised layer by layer, and the muscles surrounding the spinous processes were dissected. Laminectomy was performed at T9–T10 to expose the spinal cord; the spinal canal was opened to reveal the dura mater, and the spinous processes of T8 and T11 were clamped to stabilize the injury site. Spinal cord contusion injury was induced at the level of the T10 spinous process using an Infinite Horizons spinal impactor (IH-400: Precision Systems and Instrumentation, Lexington, Kentucky, USA). A standard mouse probe (1 mm in diameter) was used, with the impact force set to 60 kdyn (27). This force induces moderate SCI, establishing an incomplete SCI model that exhibits stable NP behaviors postoperatively. The criteria for successful model establishment were as follows: the impact site was at the midpoint of T10; paralysis of both hindlimbs occurred after awakening; and the mechanical pain threshold was below the baseline.

After the operation, the muscles and skin were sutured layer by layer, and the surgical site was disinfected with povidone-iodine to prevent infection. The mice in the sham operation group underwent laminectomy only without spinal contusion. During the anesthesia recovery period, each mouse was numbered and placed on a 30 °C constant-temperature pad until fully awake. Postoperatively, the mice were housed individually in cages. All mice received an intraperitoneal injection of 2 mL 0.9% normal saline and ceftriaxone (16 mg/kg; Zhuhai United Laboratories Co., Ltd., China) daily for 3 consecutive days post-surgery, and no signs of urinary tract infection or surgical site infection were observed throughout the study period. Manual bladder expression was performed at least twice daily until voluntary urination function was recovered.

Note: Mice that failed to meet any criteria for successful model establishment, developed severe postoperative complications or presented with unstable vital signs were excluded from the experiment. A total of 95 mice underwent surgery (including sham-operated mice) in this study, with 5 mice ultimately excluded: 3 exhibited mild injury with a postoperative BMS score > 0 and 2 had an actual impact force > 60 kdyn post-contusion. All excluded mice were replaced, yielding a final valid sample size of 90 mice. No valid experimental mice died postoperatively during the entire study period, nor were any excluded due to postoperative complications.

### 2.4 Experimental design

**Figure 1:**
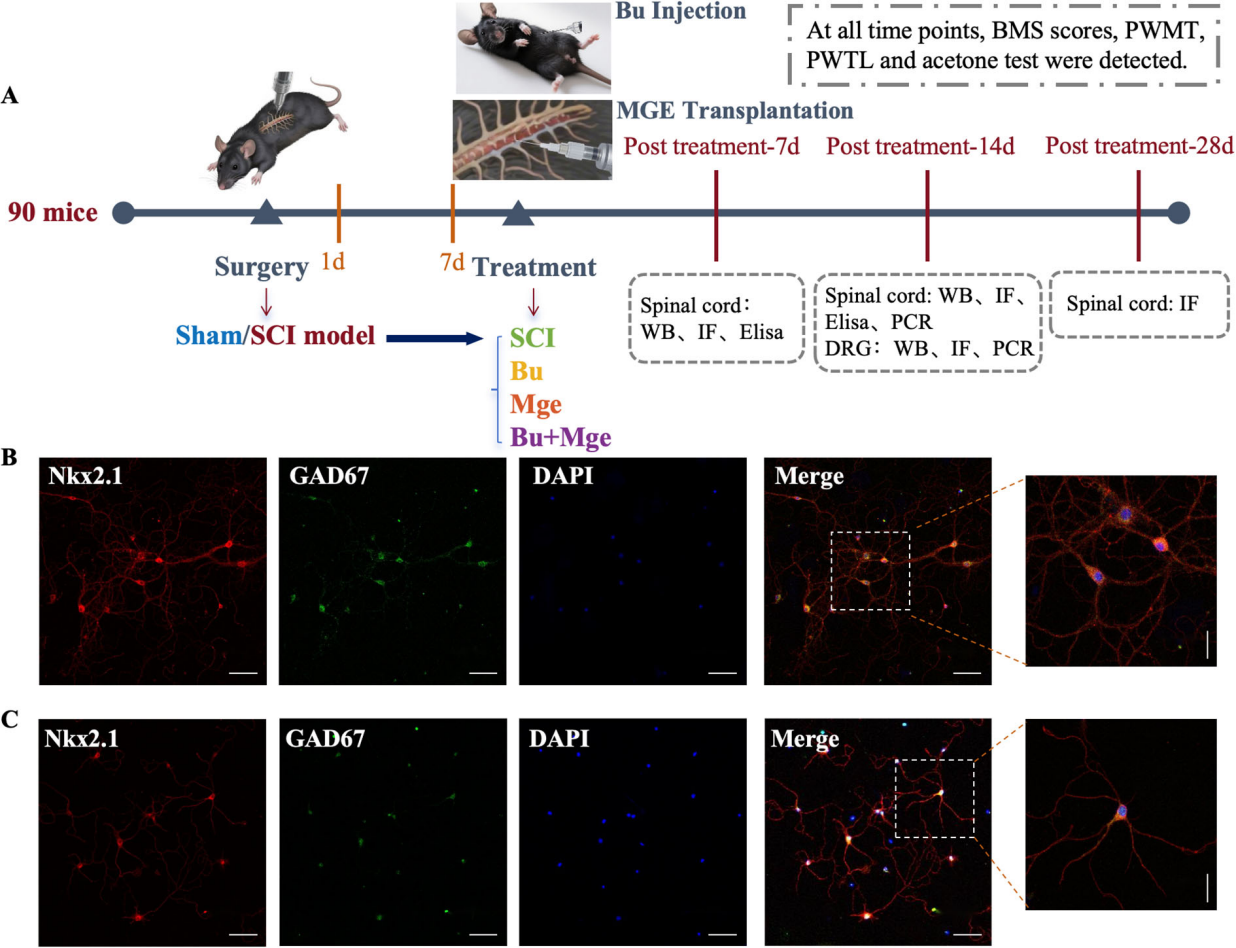
**(A)** Experimental flowchart. **(B, C)** Differentiation and identification of MGE-derived GABAergic interneuron precursor cells. **(B)** Colocalization of the precursor cell-specific marker Nkx2.1 and the GABAergic inhibitory interneuron marker GAD67 in MGE cells on day 6 of *in vitro* culture. **(C)** Colocalization of Nkx2.1 and GAD67 in MGE cells on day 11 of *in vitro* culture. Horizontal scale bar: 100 μm; Vertical scale bar: 50 μm.

A total of 90 mice were randomly divided into 5 groups with 18 mice per group using a random number table method. The experimental protocol is shown in **Figure 1A**.

- Sham group: Mice underwent T10 laminectomy without SCI;
- SCI group: Mice underwent T10 spinal cord contusion. On post-operative day 10, they received an intraperitoneal injection of an equal volume of control vehicle and transplantation of an equal volume of fibrin matrix at the site of SCI;
- Bu group: Mice underwent T10 spinal cord contusion. On post-operative day 10, they received an intraperitoneal injection of Bu and transplantation of an equal volume of fibrin matrix at the site of the SCI;
- Mge group: Mice underwent T10 spinal cord contusion. On post-operative day 10, they received an intraperitoneal injection of an equal volume of control vehicle, and transplantation of MGE cells at the site of the SCI;
- Bu + Mge combined group: Mice underwent T10 spinal cord contusion. On post-operative day 10, they received an intraperitoneal injection of Bu and transplantation of MGE cells at the site of the SCI.

### 2.5 Treatment

On post-operative day 10, an interventional treatment was administered to SCI mice with NP (28).

#### 2.5.1 MGE transplantation

After the mice were anesthetized with isoflurane through inhalation, the dorsal skin was incised to expose the spinal cord above and below the SCI level. Under a high-power microscope, the site of spinal cord contusion injury was identified; a microsyringe needle was then inserted, and 1.0 μl (5×10^4^/μl) of MGE cells was transplanted (29). The mice in the SCI group and the Bu group were transplanted with an equal volume of fibrin matrix.

#### 2.5.2 Bu injection

The NKCC1 inhibitor Bu (B129942: Aladdin, Shanghai, China) was prepared by dissolving in 10 mg/ml normal saline (0.25%) and administered at a dose of 30 mg/kg (14). The solution was administered to the mice via single intraperitoneal injection. Mice in the SCI group and MGE group received an intraperitoneal injection of an equal volume of control vehicle (normal saline containing 0.25% NaOH).

### 2.6 Behavioral assessments

All behavioral assessments in this study were performed at the same time points: before surgery, on days 1 and 10 after SCI, and on days 7, 14, and 28 post-treatment. All assessments were completed by two systematically trained researchers who were blinded to the experimental design, with the mean value of their independent evaluations calculated as the final result for each mouse to minimize subjective bias.

#### 2.6.1 Motor function assessment

The Basso Mouse Scale (BMS) was used to evaluate hindlimb motor function in mice. The BMS scores ranged from 0 to 9 points, with 0 indicating complete hindlimb paralysis and 9 indicating normal motor function.

#### 2.6.2 Paw mechanical withdrawal threshold

In accordance with a previously established pain assessment method (20), PWMT was evaluated in mice. The mice were placed in a transparent plexiglass box with a metal mesh floor and acclimated for 30 minutes. An electronic Von Frey filament algesimeter was used to apply vertical stimulation to the plantar surface of the hind paw for 6 seconds, with the stimulation force ranging from 0.07 to 1.4 g. Mechanical allodynia in the mice was assessed using the up-down paradigm to measure the paw withdrawal threshold, and the cumulative sensitivity score of the mice’s dorsum was recorded. A positive response was recorded if the mouse exhibited paw licking or withdrawal, and stimulation was continued with a lighter filament. A negative response was recorded if no paw licking or withdrawal occurred, and the process was repeated with the next heavier filament. Measurements for each mouse were performed six times or stopped when four consecutive positive or negative responses were observed. The 50% PWMT (expressed in g) was then calculated.

#### 2.6.3 Paw thermal withdrawal latency

An infrared thermal stimulator (RWD Life Technology Inc., Shenzhen, China) was used to assess thermal hyperalgesia by measuring PTWL. The mice were placed in test chambers and acclimated for 30 minutes, with a quiet environment maintained to prevent disturbance. The infrared thermal stimulus (intensity: 30%) was focused on the plantar surface of the hind paw. The instrument automatically detected hind paw lifting or licking behaviors and recorded the latency period, eliminating the need for visual scoring. Each mouse was tested 3 times, with a 5-minute interval between consecutive tests. The average value of the three measurements was taken as the PTWL. A 30-second safety cut-off time was used to prevent tissue damage (30).

#### 2.6.4 Cold hyperalgesia

The acetone test was used to assess cold hyperalgesia. After the mice were placed on a metal mesh surface and acclimated for 30 minutes, 100 μl of acetone was sprayed onto the hind paws with syringe. A total of 5 sprays were applied to the hind paw on each side, with an interval of approximately 2 minutes between consecutive sprays. Paw licking, flicking, or leg withdrawal were defined as positive responses. The total number of positive responses recorded from the 5 sprays per hind paw was converted to a percentage of the response frequency (31).

### 2.7 Collection of spinal cord and dorsal root ganglion tissue

The mice were anaesthetized via intraperitoneal injection of Avertin (20 ml/kg) after all pain behavior assessments were completed on post-treatment days 7, 14, and 28. After the chest was opened, rapid transcardial perfusion was performed via the aorta: first, perfusion with 0.9% normal saline for 2 minutes, followed by perfusion with 4% PBS. For each of the 5 groups, spinal cord tissue (0.5 cm above and below the contusion center) and DRGs from the L4–L5 and L5–L6 lumbar vertebrae were harvested. Each group contained 6 mice at each time point, among which tissues from 3 mice were used for protein related experiments, and the other 3 were used for preparing paraffin sections for IF staining. Tissues for protein experiments were immediately stored in a -80 °C freezer for long-term preservation. The tissues for paraffin sectioning were fixed in 4% paraformaldehyde at room temperature for 24 hours. Afterwards, the tissues were sequentially treated with gradient ethanol and xylene and subjected to paraffin infiltration. The spinal cord tissues were subsequently embedded in paraffin embedding medium, and the wax block was trimmed once the paraffin solidified. 6 μm-thick paraffin sections of spinal cord tissue and DRGs tissue were prepared for the following immunofluorescence staining assays, and the intact sections were air-dried before being stored at room temperature.

### 2.8 Enzyme-linked immunosorbent assay

On post-treatment days 7 and 14, the expression levels of the pain-related inflammatory factors TNF-α and IL-1β in the core region of the SCI were measured by ELISA. Spinal cord segments containing the injury center were homogenized using radioimmunoprecipitation assay (RIPA) lysis buffer (Aoqing Biotechnology Co., Ltd., Beijing, China). After homogenization, the homogenate was centrifuged at 18,600×g for 25 minutes at 4 °C. In accordance with the instructions provided with the ELISA kits (CUSABIO, Wuhan, China; Thermo Fisher Scientific, Waltham, Massachusetts, USA), a microplate reader was used to detect the absorbance values at a wavelength of 450 nm. The protein expression levels of TNF-α and IL-1β were then subsequently calculated on the basis of these absorbance values.

### 2.9 Real-time quantitative polymerase chain reaction

Total RNA from the spinal cord and DRGs was extracted using a TRNzol kit (CoWin Biotech, China) according to the manufacturer’s instructions. The nucleic acid concentration and purity were measured using a NanoDrop 2000 spectrophotometer. First-strand cDNA was synthesized using the CoWin Biotech Reverse Transcription Kit (CW2569M: Beijing, China) according to manufacturer’s protocol, and the resulting cDNA was diluted 5-fold for subsequent analysis. The PCR system (20 μl) included 400 ng of RNA template, 4 μl of dNTP mixture, 2 μl of primer mixture, 4 μl of 5× RT buffer, 2 μl of DTT, 1 μl of HiFiScript reverse transcriptase, and RNase-free water to reach the final volume. The reaction conditions were as follows: 15 minutes at 42 °C for reverse transcription, followed by 5 minutes at 85 °C to inactivate the enzyme. Relative mRNA expression levels were calculated using the 2^-^ΔΔCt method. The primers used in this study were synthesized by Sangon Biotech (Shanghai, China) and are presented in **Table 2**. By this method, the relative mRNA expression levels of key genes including glutamate decarboxylase 2 (glutamate decarboxylase 65 kDa, Gad65), glutamate decarboxylase 1 (Gad67), Slc12a2 (NKCC1) and Slc12a5 (potassium-chloride cotransporter 2, KCC2) in spinal cord and DRG tissues were quantitatively determined, with Actb (β-actin) serving as the internal reference gene for data normalization.

**Table 2:** Primers used for qRT-PCR.

Gene	Symbol	5’-3’ sequence	Reference
Glutamate decarboxylase 2	Gad65	F-TCAACTAAGTCCCACCCTAAG R-CCCTGTAGAGTCAATACCTGC	NM_008078.2
Glutamate decarboxylase 1	Gad67	F-AGGCAGTCCTCCAAGAACCT R-CCGTTCTTAGCTGGAAGCAG	NM_008077.4
NKCC1	Slc12a2	F-GGAACATTCCATACTTATGATAGATG R-CTCACCTTTGCTTCCCACTCCATTC	NM_009194.3
KCC2	Slc12a5	F-AAGGGCAGAGAGTACGATGG R-CCTGGGGTAGGTTGGTGTAG	NM_001355480.1
β-actin	Actb	F-CATTGCTGACAGGATGCAGAAGG R-TGCTGGAAGGTGGACAGTGAGG	NM_007393.5

NKCC1, Sodium-potassium-chloride cotransporter 1; KCC2, potassium-chloride cotransporter 2.

### 2.10 Western blotting

WB was used to detect the protein expression levels of inhibitor of κB (IκB), nuclear factor κB p65 subunit (P65), and TNF-α in the core region of the injured spinal cord at 7 days post-treatment. To further verify the inhibition of the NF-κB pathway, nuclear-cytoplasmic fractionation was performed on P65, and its protein expression in the nucleus and cytoplasm was detected separately. The protein expression levels of NKCC1,KCC2, and the γ-aminobutyric acid type A (GABA-A) receptor in the core region of the SCI and DRGs were also assessed at 14 days post-treatment. After total protein was extracted from the tissues, protein quantification was performed using a bicinchoninic acid (BCA) assay. Sodium dodecyl sulfate-polyacrylamide gel electrophoresis was conducted with a one-step PAGE gel rapid preparation kit (Vazyme Biotech Co., Ltd., China), and 15 μg of protein per lane was loaded for separation. The proteins were subsequently transferred onto nitrocellulose membranes (Bio-Rad, Hercules, California, USA). The membranes were blocked with 5% nonfat milk for 1 hour and then incubated overnight at 4 °C with primary antibodies against the aforementioned proteins (details are provided in **Table 1**), with β-actin serving as the internal reference protein. On the following day, the membranes were incubated with secondary antibodies (details are provided in **Table 1**) at 37 °C for 1 hour. Notably, the WB protocol for nuclear-cytoplasmic fractionation was as follows: spinal cord tissues were first homogenized with cytoplasmic extraction buffer (Beyotime Biotechnology Co., Ltd., China) to isolate cytoplasmic proteins, followed by the separation of nuclear proteins using nuclear extraction buffer (Beyotime Biotechnology Co., Ltd., China) according to the manufacturer’s instructions. The remaining operational steps were consistent with the aforementioned total protein WB procedure, with β-actin remaining as the internal reference for cytoplasmic proteins, and Histone H3 was used as the internal reference protein for nuclear fractions. The protein bands were visualized using an enhanced chemiluminescence detection system (Bio-Rad). The optical density values of the protein bands were analyzed using ImageJ 1.53k (Java 1.8.0) software developed by the National Institutes of Health (NIH, Bethesda, Maryland, USA).

### 2.11 IF staining

The prepared paraffin sections underwent xylene dewaxing and gradient ethanol rehydration. Endogenous peroxidase activity was quenched using freshly prepared 0.3% hydrogen peroxide, and antigen retrieval was performed with citrate buffer under heat. The sections were incubated overnight at 4 °C with primary antibodies (details are provided in **Table 1**). After the sections were washed with PBS, they were incubated with goat anti-rabbit IgG secondary antibodies at 37 °C for 1 hour. Following another round of washing, the sections were stained with 4’,6-diamidino-2-phenylindole (DAPI: Sakura, Torrance, California, USA) for 30 seconds. Images were captured using a Leica upright fluorescence microscope (Leica, Germany), and fluorescence intensity was analyzed using ImageJ software.

### 2.12 Statistical analysis

Data analysis was performed using SPSS 26.0 (IBM, Chicago, Illinois, USA), and graphs were generated using GraphPad Prism 10.3 (GraphPad Software, San Diego, California, USA). To ensure the detection of significant differences between groups, at least 3 animals were included in each group for *in vivo* experiments, and assessors were blinded to group assignments. For comparisons among multiple groups, one-way analysis of variance (ANOVA) was used, followed by Tukey’s *post hoc* test. For differences in functional assessment indicators (BMS scores and pain threshold results) between groups, two-way repeated-measures analysis of variance (two-way repeated-measures ANOVA) was applied, followed by Tukey’s *post hoc* test. All the data are presented as the mean ± standard error of the mean (SEM). A P-value < 0.05 was considered to indicate statistical significant. *Post-hoc* comparisons were conducted among all groups, and the results without statistical indicators marked in the figures are all non-significant differences.

## 3 Results

### 3.1 Identification of MGE

IF staining was used to assess the inducible differentiation potential of MGE cells cultured *in vitro*, with staining performed on days 6 and 11 (32, 33) of culture (**Figures 1B, C**). Nkx2.1, a transcription factor, is a stem cell-specific marker for MGE cells, whereas GAD67 is a marker of GABAergic inhibitory interneurons. As shown in **Figures 1B, C** on day 6 and 11 of *in vitro* culture, obvious colocalization of Nkx2.1 and GAD67 was observed.

### 3.2 Bu and MGE cell transplantation alone or in combination can alleviate NP-related behaviors, promote motor function recovery, and reduce the level of NP-related molecules in SCI mice

**Figure 2:**
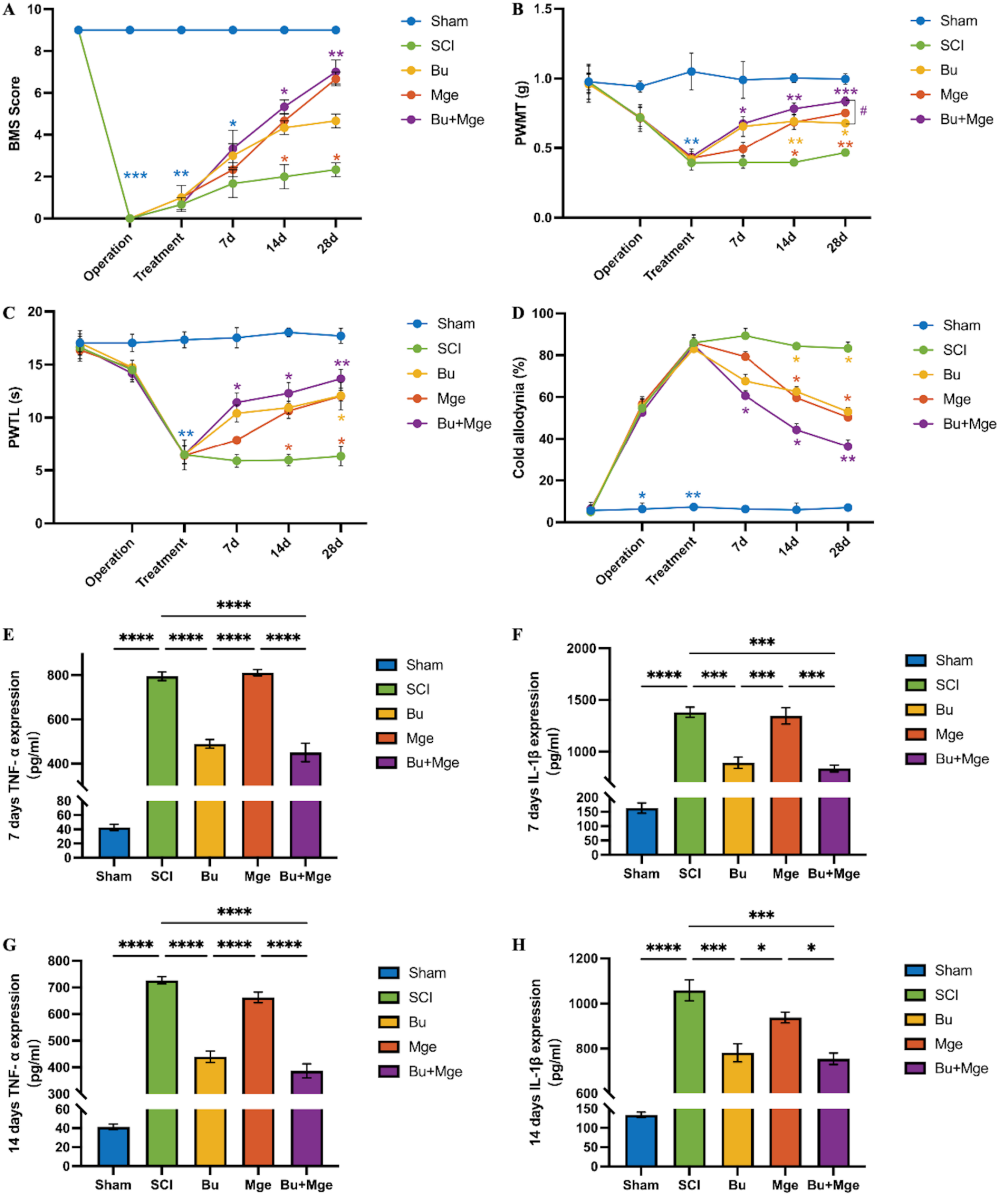
Effects of Bu injection and MGE cell transplantation alone or in combination on motor function recovery, hyperalgesia, and inflammatory factor expression levels. **(A)** Motor function recovery of the mice in each group evaluated by BMS scores (n = 6). **(B)** Mechanical allodynia in mice evaluated by PWMT. **(C)** Thermal hyperalgesia in SCI mice evaluated by PTWL. **(D)** Cold hyperalgesia in mice evaluated by the acetone test. **(E)** Changes in the expression level of the pain-related inflammatory factor TNF-α in the core region of SCI at 7 days post-treatment (n = 3). **(F)** Changes in the expression level of the pain-related inflammatory factor IL-1β in the core region of SCI at 7 days post-treatment (n = 3). **(G)** Changes in the expression level of the pain-related inflammatory factor TNF-α in the core region of the spinal cord injury at 14 days post-treatment (n = 3). **(H)** Changes in the expression level of the inflammatory factor IL-1β in the core region of the spinal cord injury at 14 days post-treatment (n = 3). The data are presented as the mean ± SEM. *p < 0.05, **p < 0.01, ***p < 0.001, ****p < 0.0001, #p < 0.05 (Bu+MGE vs. Bu group).

As shown in **Figures 2A**, after SCI, the BMS scores of all four groups (excluding the Sham group) were 0; the scores tended to increase over time. Among these groups, the BMS scores of the Bu+Mge and Mge groups were greater than those of the SCI group at each time point starting from day 14 post-treatment (p < 0.05).

NP after SCI was evaluated by measuring the PWMT, PTWL, and though the acetone test before and after treatment (**Figures 2B–D**). After SCI, the mice exhibited mechanical, thermal, and cold hyperalgesia. Before treatment, the relevant indicators of the four surgical groups showed significant differences compared with those of the Sham group (p < 0.01), confirming the successful establishment of the SCI-NP mouse model. Starting on day 7 post-treatment, the Bu+Mge group exhibited significant improvements in PWMT, PTWL, and cold hyperalgesia compared with those of the SCI group (p < 0.05). On day 28 post-treatment, the therapeutic effect on PWMT in the Bu+Mge group was significantly greater than that in the Bu group (p = 0.035). The mechanical allodynia and cold hyperalgesia of the Bu and Mge groups improved starting from day 14 post-treatment (p < 0.05). With respect to PTWL, the therapeutic effects in the Bu group started on day 28 post-treatment (p < 0.05), whereas those of the Mge group started on day 14 post-treatment (p < 0.05).

To further investigate the changes in inflammatory responses during NP alleviation by Bu injection and MGE cell transplantation, the expression levels of TNF-α and IL-1β were determined. The ELISA results (**Figures 2E–H**) revealed that the expression levels of pain-related inflammatory factors (TNF-α, p < 0.0001; IL-1β, p < 0.001) decreased to varying degrees in the Bu and Bu+Mge groups on days 7 and 14 post-treatment.

### 3.3 Effects of Bu alone and in combination with MGE cell transplantation on NF-κB pathway activation

**Figure 3:**
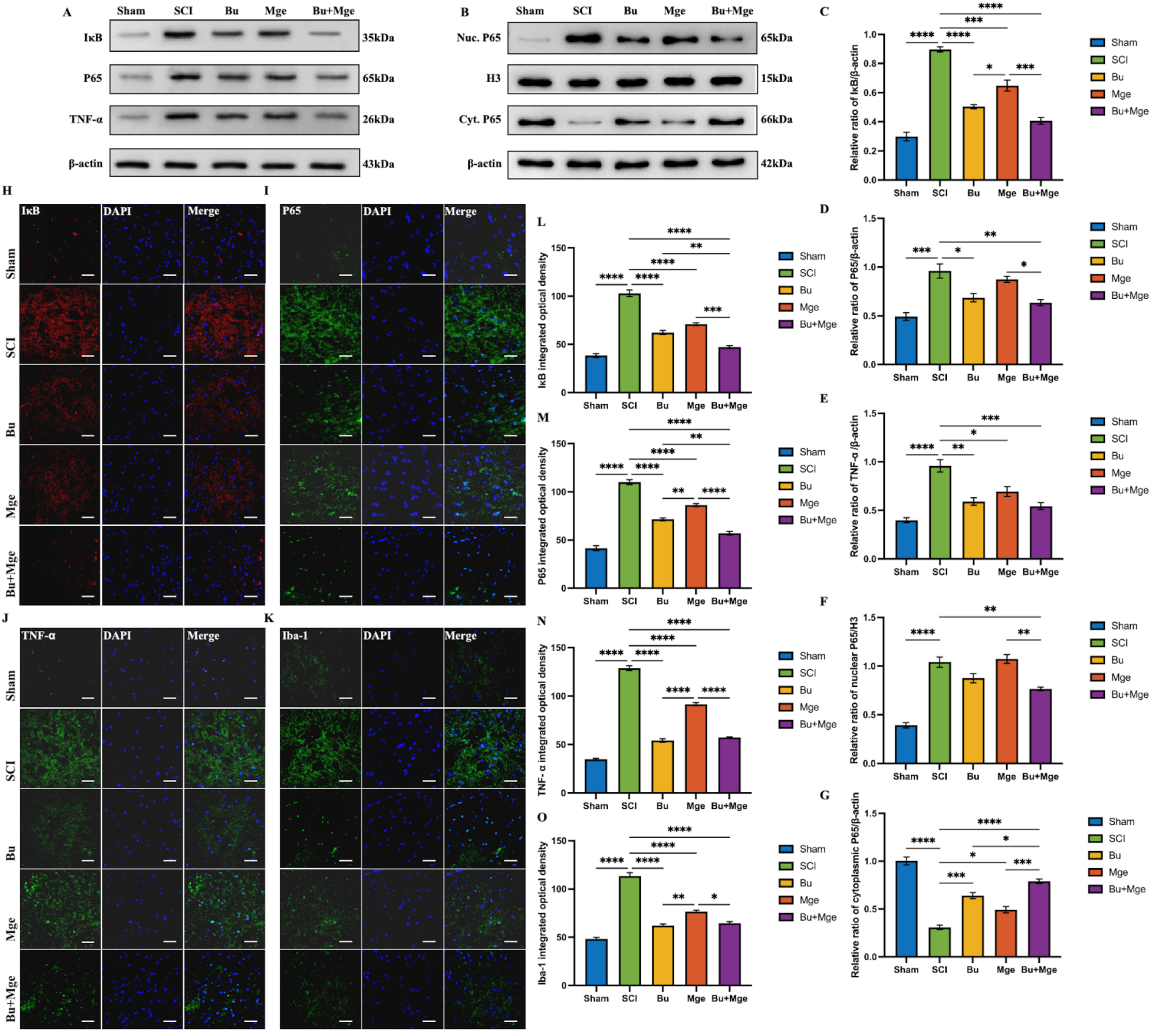
Effects of Bu, MGE cell transplantation, and their combination on the NF-κB pathway at 7 days post-treatment. **(A)** Representative WB images of spinal cord tissue. **(B)** Representative image of P65 WB after cytoplasmic/nuclear fractionation. **(C–E)** Semi-quantitative results of IκB, P65, and TNF-α proteins expression by WB (n = 3). **(F, G)** Semi-quantitative results of P65 protein expression after cytoplasmic/nuclear fractionation by WB (n = 3). **(H–K)** IF staining of IκB, P65, TNF-α, and Iba-1. **(L–O)** Semi-quantitative analysis of IκB, P65, TNF-α, and Iba-1 expression by IF staining (n = 3). Scale bar: 50 μm. The data are presented as the mean ± SEM. *p < 0.05, **p < 0.01, ***p < 0.001, ****p < 0.0001.

On day 7 post-treatment, the effects of Bu, MGE cell transplantation, and their combination on the NF-κB pathway were verified. WB results (**Figures 3A–G**) revealed that, compared with that in the SCI group, the expression of IκB in the treatment groups decreased (p < 0.001); among these, significant differences in IκB expression were also observed between the Bu/Bu+Mge group and the Mge group (p < 0.05). The expression of P65 in the Bu and Bu+Mge groups was also significantly lower than that in the SCI group (p < 0.05). Although the expression of P65 in the Mge group was lower than that in the SCI group, the difference was not statistically significant, the WB results of the cytoplasmic/nuclear fractionation experiment further confirm this point. The expression of TNF-α in all three treatment groups was lower than that in the SCI group (p < 0.05), but no significant differences were found among the different treatment groups. The expression trends of IκB and P65 determined by IF staining were generally consistent with those determined by WB (**Figures 3H–J, L–N**). However, the expression levels of IκB and P65 in the Bu+Mge group were significantly lower than those in the other treatment groups (p < 0.01). The expression of ionized calcium-binding adapter molecule 1 (Iba-1), a microglial marker, decreased after treatment (**Figures 3K, O**); specifically, the expression levels in the Bu and Bu+Mge groups were significantly lower than those in the Mge group (p < 0.05). The IF staining results for TNF-α were consistent with this trend.

### 3.4 Bu and MGE cell transplantation alone or in combination can affect the expression of NKCC1 and KCC2 in spinal cord tissue

**Figure 4:**
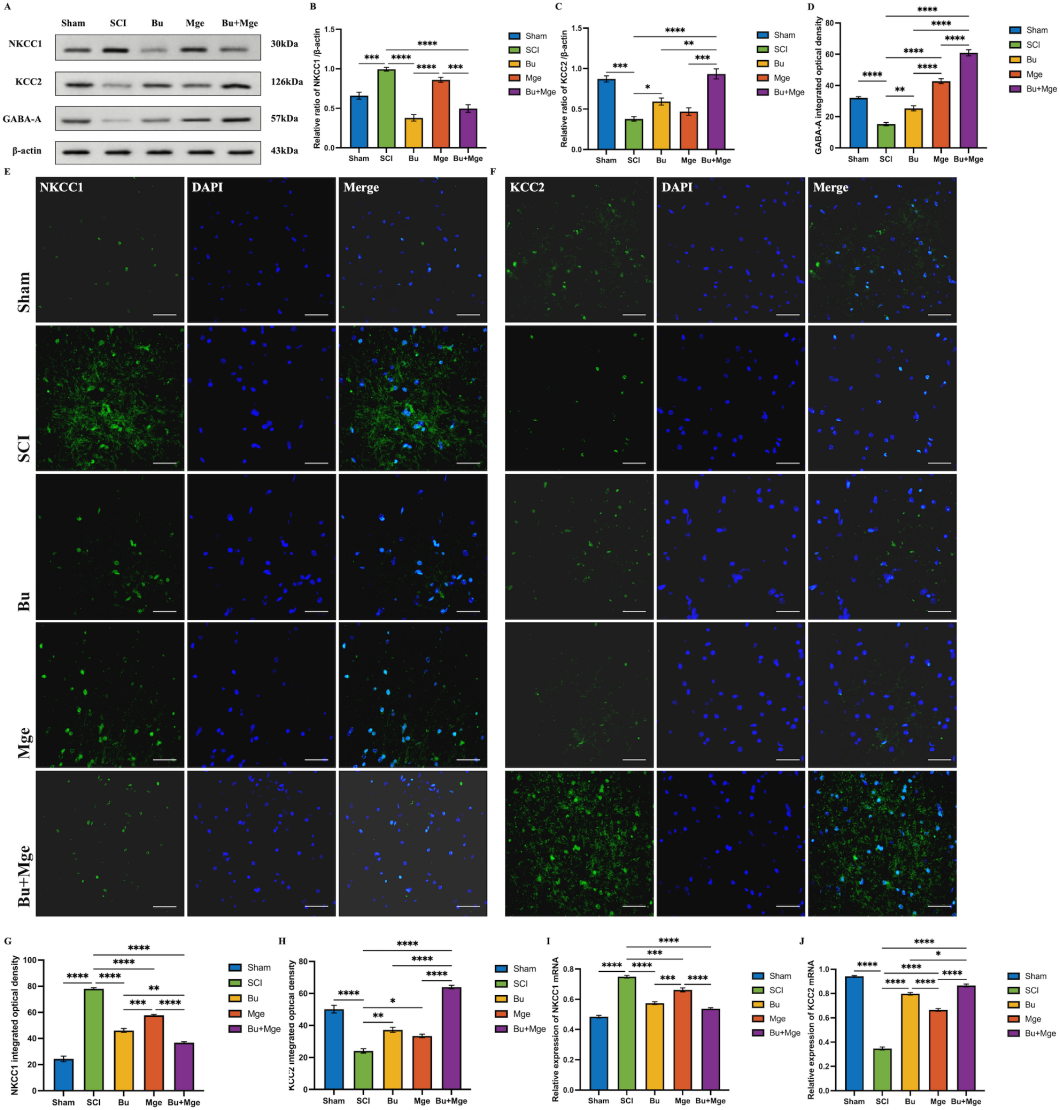
Effects of Bu, MGE cell transplantation, and their combination on the expression of NKCC1, KCC2 and GABA-A receptor in spinal cord tissue at 14 days post-treatment. **(A)** Representative WB images of spinal cord tissue. **(B-D)** Semi-quantitative results of NKCC1, KCC2 and GABA-A receptor proteins expression by WB (n = 3). **(E)** IF staining of NKCC1. **(F)** IF staining of KCC2. **(G)** Semi-quantitative analysis of the IF staining for expression of NKCC1 (n = 3). **(H)** Semi-quantitative analysis of the IF staining for expression of KCC2 (n = 3). **(I)** mRNA expression level of the NKCC1 gene measured by qRT-PCR (n = 3). **(J)** mRNA expression level of the KCC2 gene measured by qRT-PCR (n = 3). Scale bar: 50 μm. The data are presented as the mean ± SEM. *p < 0.05, **p < 0.01, ***p < 0.001, ****p < 0.0001.

On day 14 post-treatment, the effects of Bu, MGE cell transplantation, and their combination on the expression levels of NKCC1 and KCC2 in spinal cord tissue were investigated. WB results (**Figures 4A–C**) revealed that after SCI, the expression level of NKCC1 significantly increased (p < 0.001) but decreased after treatment. Specifically, the Bu and Bu+Mge groups presented significant differences in NKCC1 expression compared with that of the SCI group (p < 0.0001) and that of the Mge group (p < 0.001); however, there was no significant difference between the Mge and SCI groups. An increase in NKCC1 expression was accompanied by suppressed KCC2 expression. After treatment, the KCC2 expression level increased with the Bu and Bu+Mge groups exhibiting significant differences when compared with the SCI group (p < 0.05), and the combination treatment group exhibited a significant difference when compared with the monotherapy groups (p < 0.01). The IF staining results (**Figures 4E–H**) were generally consistent with those of WB, with the combination therapy group showing the most significant improvement in all the indicators (p < 0.0001). This trend was further confirmed by qRT-PCR analysis of the mRNA expression of the NKCC1 and KCC2 genes (**Figures 4I, J**).

### 3.5 Bu and MGE cell transplantation alone or in combination can affect the expression of NKCC1 and KCC2 in DRG tissue

**Figure 5:**
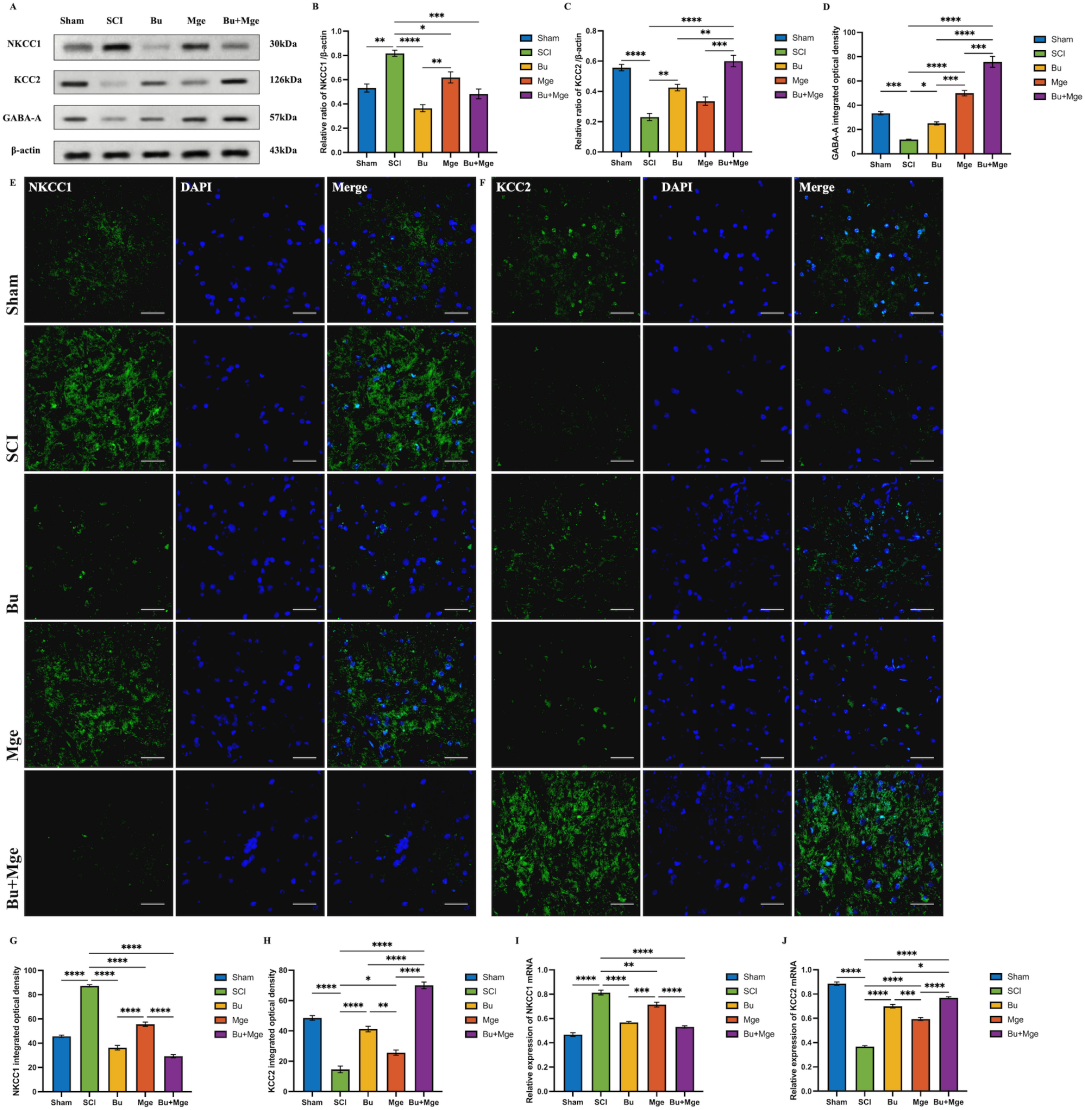
Effects of Bu, MGE cell transplantation, and their combination on the expression of NKCC1, KCC2 and GABA-A receptor in DRG tissue at 14 days post-treatment. **(A)** Representative WB images of DRG tissue. **(B-D)** Semi-quantitative results of NKCC1, KCC2 and GABA-A receptor proteins expression by WB (n = 3). **(E)** IF staining of NKCC1. **(F)** IF staining of KCC2. **(G)** Semi-quantitative analysis of the IF staining for expression of NKCC1 (n = 3). **(H)** Semi-quantitative analysis of the IF staining for expression of KCC2 (n = 3). **(I)** mRNA expression level of the NKCC1 gene measured by qRT-PCR (n = 3). **(J)** mRNA expression level of the KCC2 gene measured by qRT-PCR (n = 3). Scale bar: 50 μm. The data are presented as the mean ± SEM. *p < 0.05, **p < 0.01, ***p < 0.001, ****p < 0.0001.

On day 14 post-treatment, the effects of Bu, MGE cell transplantation, and their combination on the expression levels of NKCC1 and KCC2 in DRG tissue were detected. WB results (**Figures 5A–C**) revealed that after SCI, the expression level of NKCC1 significantly increased (p < 0.001) but decreased after treatment (p < 0.05 compared with that in the SCI group); notably, a significant difference in NKCC1 expression was observed between the Bu and Mge groups (p < 0.01). The expression level of KCC2 increased after treatment: compared with the SCI group, the Bu and Bu+Mge groups showed significant differences (p < 0.05), and an additional significant difference was found between the Bu+Mge group and the Bu group (p < 0.01). The IF staining results (**Figures 5E–H**) were generally consistent with those of the WB, with the combination treatment group showing the most significant improvement in all indicators (p < 0.0001). This trend was further confirmed by qRT-PCR analysis of the mRNA expression levels of the NKCC1 and KCC2 genes (**Figures 5I, J**).

### 3.6 MGE cell transplantation alone or in combination affects the expression of GABAergic-related markers in mice

**Figure 6:**
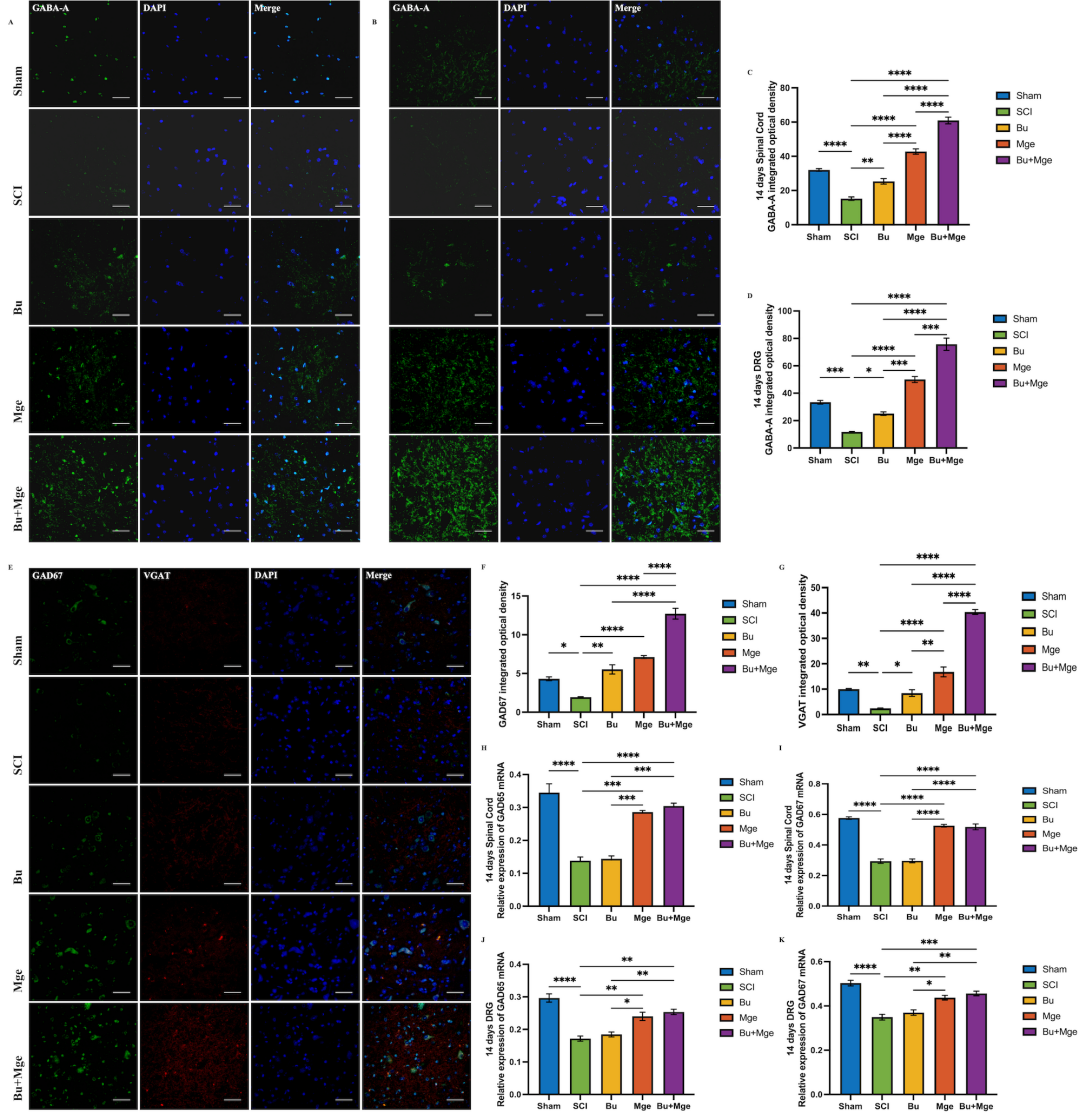
MGE cell transplantation alone or in combination affects the expression of GABAergic-related markers in mice. **(A)** IF staining of GABA-A receptor in spinal cord tissue at 14 days post-treatment. **(B)** Semi-quantitative analysis of the IF staining for expression of GABA-A receptor in spinal cord tissue at 14 days post-treatment (n = 3). **(C)** IF staining of GABA-A receptor in DRG at 14 days post-treatment. **(D)** Semi-quantitative analysis of the IF staining for expression of GABA-A receptor in DRG at 14 days post-treatment (n = 3). **(E)** IF staining of GAD67 and VGAT in spinal cord tissue at 28 days post-treatment. **(F, G)** Semi-quantitative analysis of the IF staining for expression of GAD67 and VGAT. (n = 3) **(H)** GAD65 mRNA expression level in spinal cord tissue at 14 days post-treatment (n = 3); **(I)** GAD67 mRNA expression level in spinal cord tissue at 14 days post-treatment (n = 3); **(J)** GAD65 mRNA expression level in DRG tissue at 14 days post-treatment (n = 3); **(K)** GAD67 mRNA expression level in DRG tissue at 14 days post-treatment (n = 3). Scale bar: 50 μm. The data are presented as the mean ± SEM. *p < 0.05, **p < 0.01, ***p < 0.001, ****p < 0.0001.

The effects of Bu, MGE cell transplantation, and their combination on the expression levels of GABAergic-related markers in spinal cord or DRG tissue were detected. WB results at 14 days post-treatment (**Figures 4A, D** and **5A, D**) revealed that the expression levels of GABA-A receptors were significantly increased in both the spinal cord and DRG of the MGE group and the Bu+MGE group compared with those in the SCI group (p < 0.01), whereas the expression of GABA-A receptors in the Bu group was not obviously affected. Consistently, the IF staining results (**Figures 6A–D**) of GABA-A receptors in spinal cord tissue and DRG at 14 days post-treatment were generally consistent with those of WB, and the combination treatment group showed the most significant improvement (p < 0.0001). In addition, double-labeled IF staining results (**Figures 6E–G**) of GAD67 (a core marker of GABAergic neurons) and vesicular GABA transporter (VGAT) in spinal cord tissue at 28 days post-treatment indicated that both MGE alone and in combination significantly upregulated the expression of GABAergic-related markers (p < 0.0001); notably, the expression level of these markers in the Bu+MGE group also showed a significant difference compared with that in the MGE group (p < 0.0001). qRT-PCR was used to analyze the mRNA expression levels of the GAD65 and GAD67 genes, key biosynthetic enzymes for GABA, on day 14 post-treatment (**Figures 6H–K**). The results revealed that in both spinal cord and DRG tissue, MGE cell transplantation alone or in combination significantly increased the mRNA expression levels of GAD in mice (p < 0.01). In contrast, Bu monotherapy had no effect on GAD mRNA expression.

### 3.7 Bu and MGE cell transplantation alone or in combination, promote nerve repair in SCI mice and reduce glial scar formation

**Figure 7:**
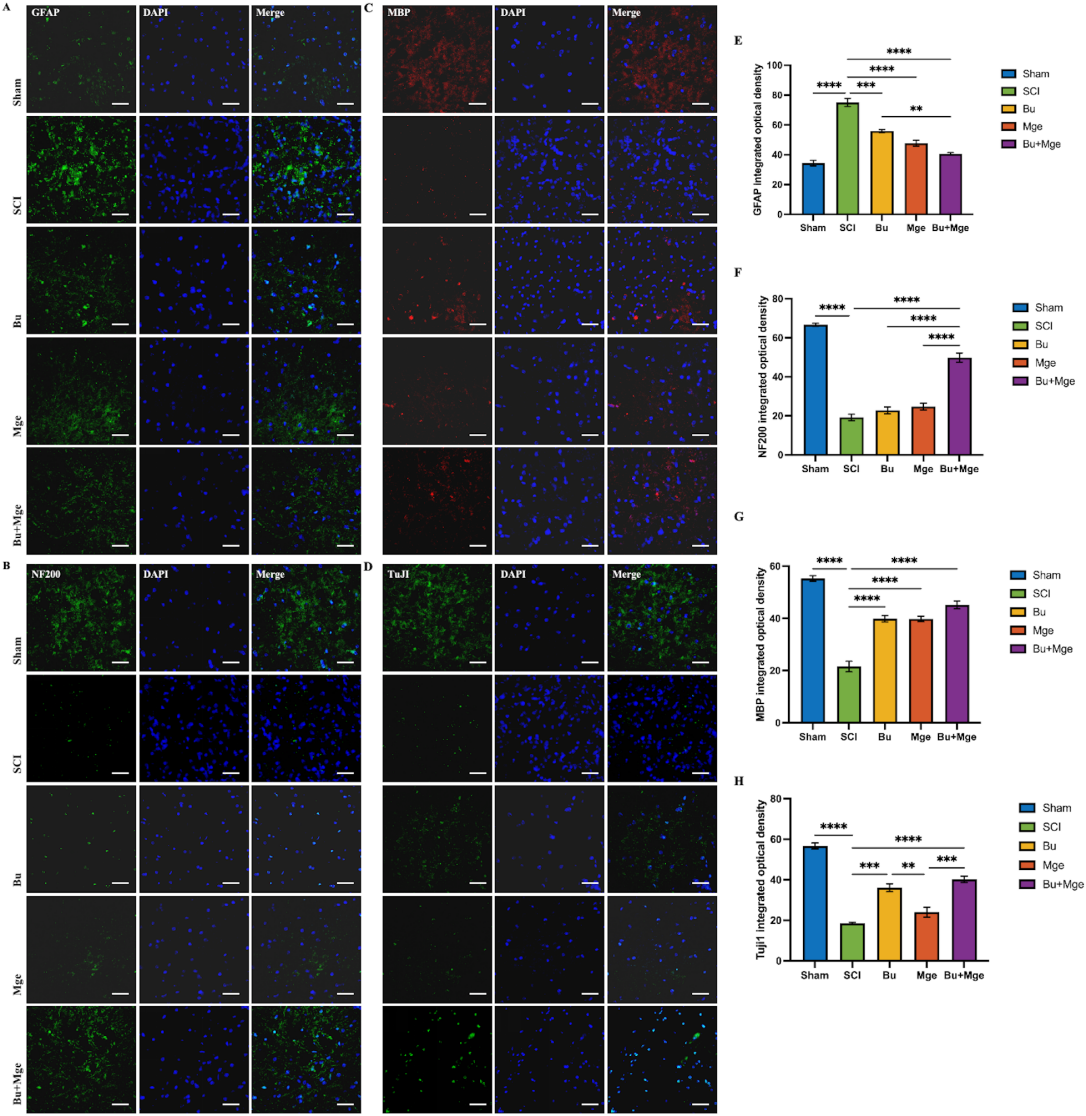
Bu and MGE cell transplantation alone or in combination therapy promoted neural repair and reduced glial scar formation in mice with SCI. **(A–D)** IF staining of GFAP, NF200, MBP and Tuj1 in spinal cord tissue at 28 days post-treatment; **(E–H)** Semi-quantitative analysis of GFAP, NF200, MBP and Tuj1 expression in spinal cord tissue at 28 days post-treatment (n = 3). Scale bar: 50 μm. The data are presented as the mean ± SEM. *p < 0.05, **p < 0.01, ***p < 0.001, ****p < 0.0001.

IF staining results at 28 days post-treatment revealed that the expression of the astrocyte marker glial fibrillary acidic protein (GFAP) was significantly increased after injury (**Figures 7A, E**). Treatment attenuated astrocyte activation and reduced glial scar formation (p < 0.01), with a significant difference observed between the Bu+Mge group and the Bu group (p < 0.01). Neurofilament loss was evident after SCI (**Figures 7B, F**), and treatment reduced this degree of neurofilament loss (p < 0.0001). The expression of the neuronal marker β-tubulin III (Tuj1) significantly decreased after injury (**Figures 7D, H**), whereas the combination treatment with Bu and MGE protected neurons (p < 0.0001). Myelin basic protein (MBP) expression was significantly reduced in the SCI group (**Figures 7C, G**), and both the Bu monotherapy and the Bu+Mge combination treatment protected myelin (p < 0.001).

## 4 Discussion

This study provides evidence for the alleviation of NP after SCI by Bu, MGE cell transplantation, and their combination. All three therapeutic approaches effectively reduced hyperalgesia symptoms in SCI mice while promoting the recovery of motor and neurological functions in the mice. The underlying mechanisms involve the following aspects: Bu inhibits neuroinflammation by regulating the activation of the NF-κB pathway, thereby reducing the occurrence of NP in the early stage of treatment; Bu modulates the expression of NKCC1 and KCC2 in the spinal cord and DRGs by regulating Cl^-^ homeostasis, which effectively improves NP; and MGE alleviates NP symptoms by increasing the inhibitory effect of GABAergic neurons.

### 4.1 The combination therapy is more effective in improving NP than the monotherapy is

This study demonstrated that Bu injection, MGE cell transplantation, or their combination alleviated hyperalgesia, promoted motor function recovery, and enhanced neural repair in SCI mice, with combination therapy showing the most significant effects. BMS scores indicated that MGE and combination therapy accelerated motor function recovery, likely due to GABAergic neuron-mediated repair of damaged motor circuits (34). For NP, combination therapy alleviated hyperalgesia from post-treatment day 7, earlier than monotherapies, and exerted stronger effects on mechanical hyperalgesia than Bu monotherapy, confirming its rapid and effective analgesic role in SCI-NP. Previous studies have confirmed that Bu, an NKCC1 inhibitor, can effectively improve NP in animal models of SCI (35), peripheral nerve injury (PNI) (36), and traumatic brain injury (TBI) (37). Additionally, another study (18) reported that MGE transplantation into the mouse spinal cord can alleviate NP caused by nerve injury. However, there are currently no reports on the efficacy of the combination of these two interventions.

TNF-α and IL-1β released by activated microglia are key factors that mediate secondary inflammatory injury and pain sensitization after nerve injury (38). In this study, the ELISA results confirmed that Bu monotherapy or combination therapy could significantly inhibit the expression of TNF-α and IL-1β, whereas MGE transplantation had a minimal effect on improving the expression of inflammatory factors. This result is consistent with the previously reported conclusion that MGE does not improve inflammatory pain (18). This finding suggests that Bu exerts an early analgesic effect by directly inhibiting the inflammatory response (39). Although the combination therapy did not further reduce the levels of inflammatory factors, the subsequent analysis of GABAergic neuron markers indicated that it achieved superior pain relief by restoring the inhibitory function of GABAergic neurons.

In the middle to late stages following SCI, glial scars impede neural regeneration and neurological recovery (40). The IF staining at post-treatment day 28 revealed lower expression of GFAP, a glial scar marker, in the combination therapy group than in the Bu group, indicating that combination therapy more effectively inhibits astrocyte activation and reduces glial scar formation. Moreover, the loss of the axonal structural protein neurofilament 200 kDa protein (NF200) and Tuj1 was minimal in the combination therapy group. This finding indicates that combination therapy can significantly protect neurons and axons, providing a structural basis for the recovery of motor function (41). In addition, the expression of MBP was significantly increased in both the Bu monotherapy and combination therapy groups, suggesting that Bu can maintain neural signal transmission by protecting myelin sheaths (42) and that MGE cell transplantation can further increase this protective effect. Therefore, this study revealed that combination therapy of Bu and MGE can achieve long-term neural repair after SCI through multiple mechanisms, including inhibiting scar formation, protecting neurons, and preserving myelin sheaths. This conclusion was consistent with the continuously increasing BMS scores.

### 4.2 Bu-mediated inhibition of the NF-κB pathway is the core anti-inflammatory mechanism underlying the synergistic effect of combination therapy

The NF-κB pathway is involved in the occurrence and development of inflammatory responses and NP. Therefore, inhibiting the activation of the NF-κB pathway is a potential therapeutic strategy for pain relief (43). As a key inhibitory protein in the NF-κB signaling pathway, IκB is widely present in the cytoplasm of eukaryotic cells. It maintains the quiescent state of NF-κB by binding to it and inhibiting its nuclear translocation, thereby regulating physiological processes such as immunity and inflammation (44). After SCI, IκB undergoes phosphorylation and degradation, releasing the P65 subunit of NF-κB to translocate into the nucleus and initiating the transcription of pro-inflammatory factors such as TNF-α and IL-1β (45). The results of the WB and IF staining assays in this study revealed that both Bu monotherapy and combination therapy directly inhibited IκB degradation and P65 nuclear translocation, effectively blocking the activation of the NF-κB pathway. This effect was observable as early as 7 days post-treatment.

Microglia are key effector cells for the release of pro-inflammatory mediators following central nervous system injury (46). The activation of microglia and the interaction between neurons and glial cells are important mechanisms underlying the occurrence of neuroinflammation and the development of central sensitization, and they play a crucial role in the initiation and maintenance of the NP (47). Assessment of the expression level of the microglial marker Iba-1 in the spinal cord at 7 days post-treatment through IF staining revealed that both Bu monotherapy and combination therapy significantly and effectively inhibited microglial activation, thereby further alleviating microglia-mediated inflammatory damage.

Notably, MGE cell transplantation did not significantly inhibit the activation of either microglia or the NF-κB pathway. On the basis of the previous results of inflammatory factor level assessment and the behavioral data regarding NP improvement in the early treatment stage, it can be inferred that Bu primarily alleviates NP by exerting anti-inflammatory effects, whereas MGE cell transplantation lacks such anti-inflammatory and analgesic effects.

### 4.3 Restoring GABAergic inhibitory function is the key to improving neurofunction by combination therapy

SCI-induced disruption of GABAergic inhibitory circuits contributes to NP and neuronal hyperexcitability (10), which depends on Cl^-^ homeostasis regulated by NKCC1 and KCC2. Numerous studies have confirmed that following the onset of NP, the expression of NKCC1, a key mediator of Cl^-^ accumulation in primary nociceptive neurons, increases significantly (11, 14, 48). This increase in turn leads to downregulated KCC2 expression and elevated intracellular Cl^-^ concentrations, which disrupt the anion homeostasis of GABAergic neurons. Consequently, synaptic currents that originally exert inhibitory effects are converted into excitatory currents, triggering pathological neuronal excitation and further exacerbating pain sensitization. Previous studies have shown that pain can be effectively alleviated through pharmacological blockade or genetic knockout of NKCC1 (49, 50). This study further validated this conclusion through WB and IF analyses of injured spinal cord and DRG tissues. The results from this study demonstrated that Bu specifically inhibited NKCC1 to directly rectify the imbalance of Cl^-^ transporters, reducing intracellular Cl^-^ concentrations to below their equilibrium potential. This provides a microenvironmental basis for GABA to restore its inhibitory function, thereby alleviating NP symptoms. Although MGE cell transplantation fails to improve Cl^-^ homeostasis, it relies instead on the additive effect with Bu to achieve therapeutic benefits.

The enhancement of GABAergic neuron function in local spinal neural pathways is one of the potential mechanisms for altering the NP. MGE cells transplanted into the injured spinal cord can differentiate into GABAergic neuron subtypes *in vivo* (51, 52) and can functionally integrate into local functional electrical conduction circuits, thereby exerting an inhibitory effect. In this study, GABAergic progenitor cells, specifically MGE cells extracted from the brains of fetal mice, were transplanted into the *in situ* injured spinal cord on day 10 post-SCI and the results revealed that the expression of GABA-A receptors and other GABAergic-related markers in the treatment group that received MGE cell transplantation was significantly greater than that in the SCI group. Thus, MGE cell transplantation may achieve analgesic effects by differentiating into functional GABAergic interneurons and integrating into host circuits, which is consistent with the previous findings of Asiedu et al. (53).

Neural injury downregulates GAD,a key GABA biosynthetic enzyme (54). The qRT-PCR revealed that the GAD65/67 mRNA levels in the transplantation group showed no significant differences with those of the Sham group. This result suggests that MGE transplantation does not simply upregulate GAD mRNA expression, but rather alleviates the hyperexcitability of GABAergic neurons by counteracting the reduction in GAD mRNA caused by neural injury, which is consistent with previous research conclusions (54). MGE’s analgesic effect relies on differentiation into GABAergic neurons, which requires at least 2 weeks *in vivo* to acquire a mature neuronal phenotype and respond to peripheral stimuli (18). This time point is highly consistent with the time when a significant difference in pain threshold emerged between the MGE monotherapy group and the SCI group, further confirming the rationality of the aforementioned mechanism.

Bu can correct the imbalance of Cl^-^ homeostasis in nerve cells, whereas MGE cells supplement functional GABAergic inhibitory effects by differentiating into GABAergic neurons. When these two interventions are used in combination, the low Cl^-^ microenvironment established by Bu can significantly amplify the normal inhibitory neurotransmission function mediated by GABAergic neurons differentiated from MGE cells, ultimately forming an additive mechanism characterized by both homeostasis correction and increased inhibitory function. This is precisely the core reason why the combination therapy group was superior to the monotherapy group in alleviating NP symptoms and promoting the recovery of motor function.

## 5 Limitation

Although this study confirmed the efficacy and underlying mechanism of the combination therapy of Bu and MGE cells, it still had the following limitations. First, direct detection of the synaptogenesis of GABAergic neurons was lacking. For instance, recording GABAergic postsynaptic currents via the patch-clamp technique could directly confirm the restoration of GABA inhibitory function. Second, an in-depth mechanistic investigation of the regulatory effects of key effector molecules secreted by MGE cells on inflammation and the GABA pathway was lacking, and further verification through transcriptome sequencing or proteomics is needed. Third, this study has a small sample size; although basic statistical power was ensured based on 3R principles and pre-experiments, the small sample size may reduce the stability and generalizability of the results, and future studies should expand the sample size to further verify the mechanism of the combined therapy in this study. In addition, this study used only female mice, and the potential influence of sex as a biological variable cannot be overlooked. Previous studies (55, 56) have confirmed sexual dimorphism in pain pathways, with core disparities in peripheral receptor coding, spinal dorsal horn neuroimmune mechanisms, brain network processing, and hormone-gene regulation. Future studies should incorporate male mice to clarify sex-dependent efficacy of the combination therapy, thus providing more comprehensive experimental evidence for clinical individualized treatment.

## 6 Conclusion

This study confirms that the combination therapy of Bu and MGE cell transplantation can additively improve NP and motor function in SCI mice through multiple mechanisms: i) inhibiting the activation of the NF-κB pathway and microglia to alleviate inflammatory damage; ii) correcting the imbalance of NKCC1/KCC2 in the spinal cord and DRGs to restore GABA inhibitory function; iii) increasing the expression of GAD65/67 mRNA and GABA-A receptors to enhance the GABAergic inhibitory circuit; iv) reducing glial scar formation to protect neurons, axons, and myelin sheaths. The combination therapy has the core complementary advantages of correcting chloride ion homeostasis and enhancing GABAergic function, providing a new theoretical basis and potential strategies for the clinical treatment of NP following SCI.

## Data Availability

The original contributions presented in the study are included in the article/**Supplementary Material**. Further inquiries can be directed to the corresponding author.
