# Supplementary material for: Combined use of Bumetanide and MGE cell transplantation alleviates neuropathic pain and its mechanism after spinal cord injury in mice
Source: Front Immunol. 2026 Mar 18;17:1751436. doi: 10.3389/fimmu.2026.1751436 (PMC13038438; doi:10.3389/fimmu.2026.1751436)

Figure 3A

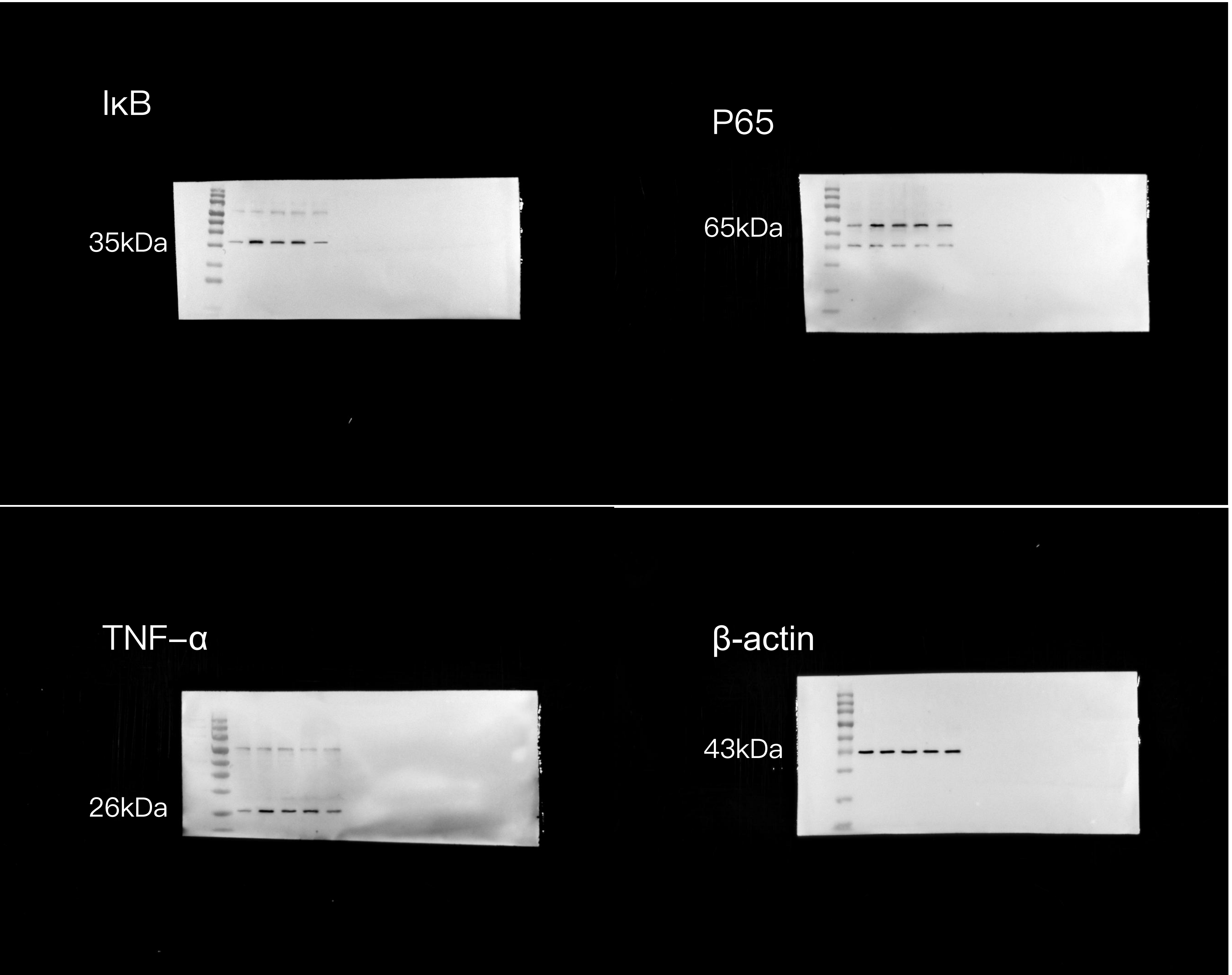

Figure 3B

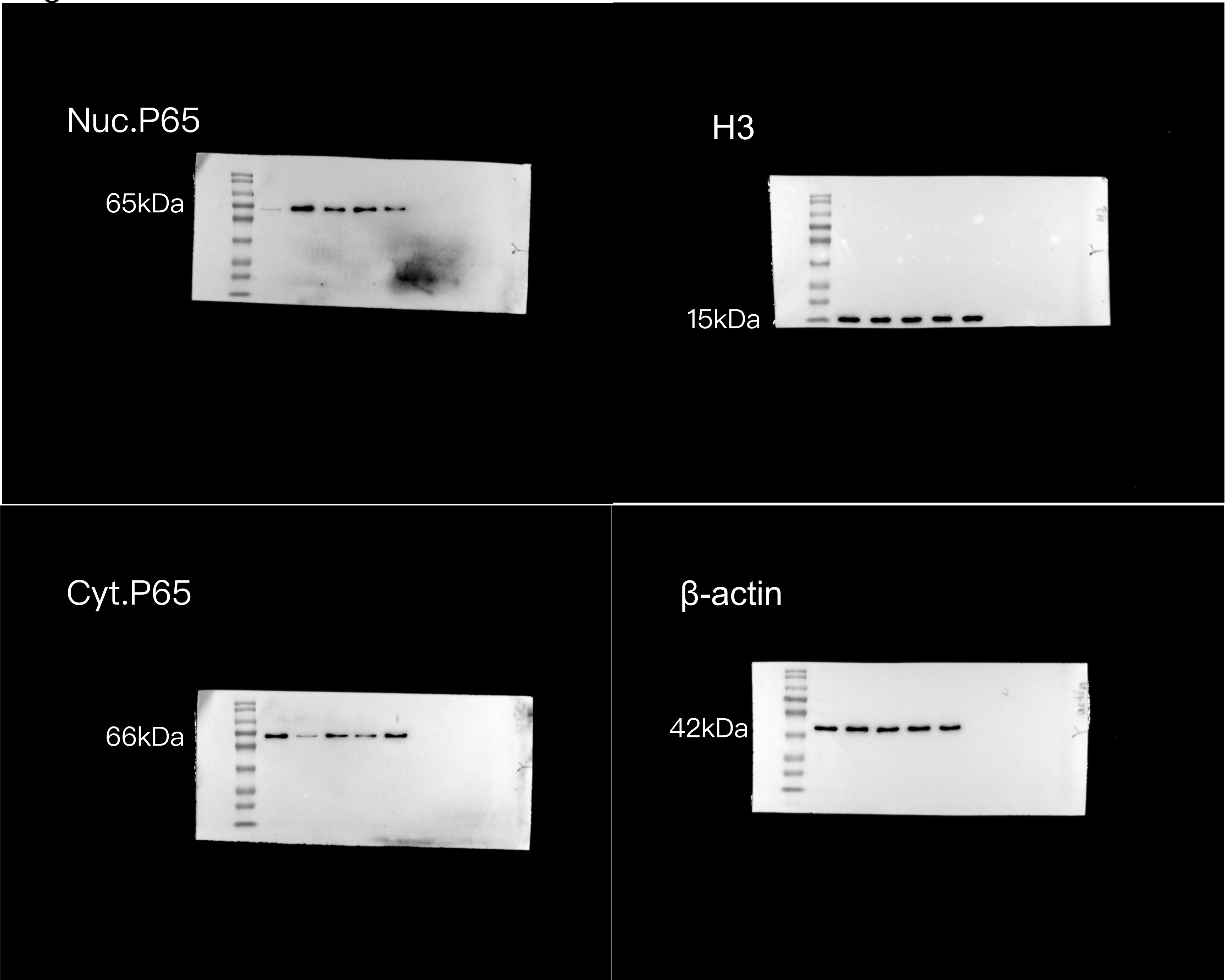

Figure 4A

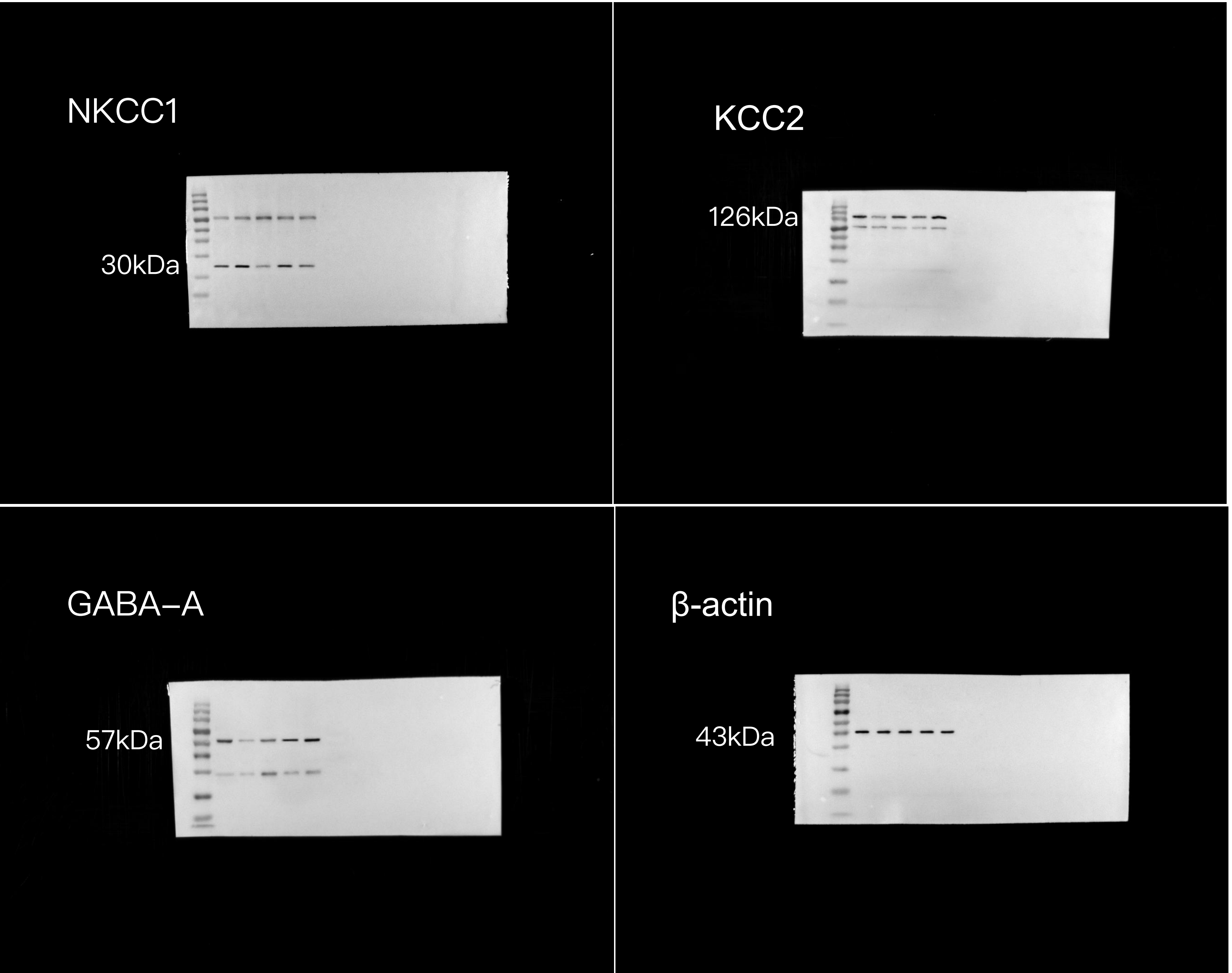

Figure 5A

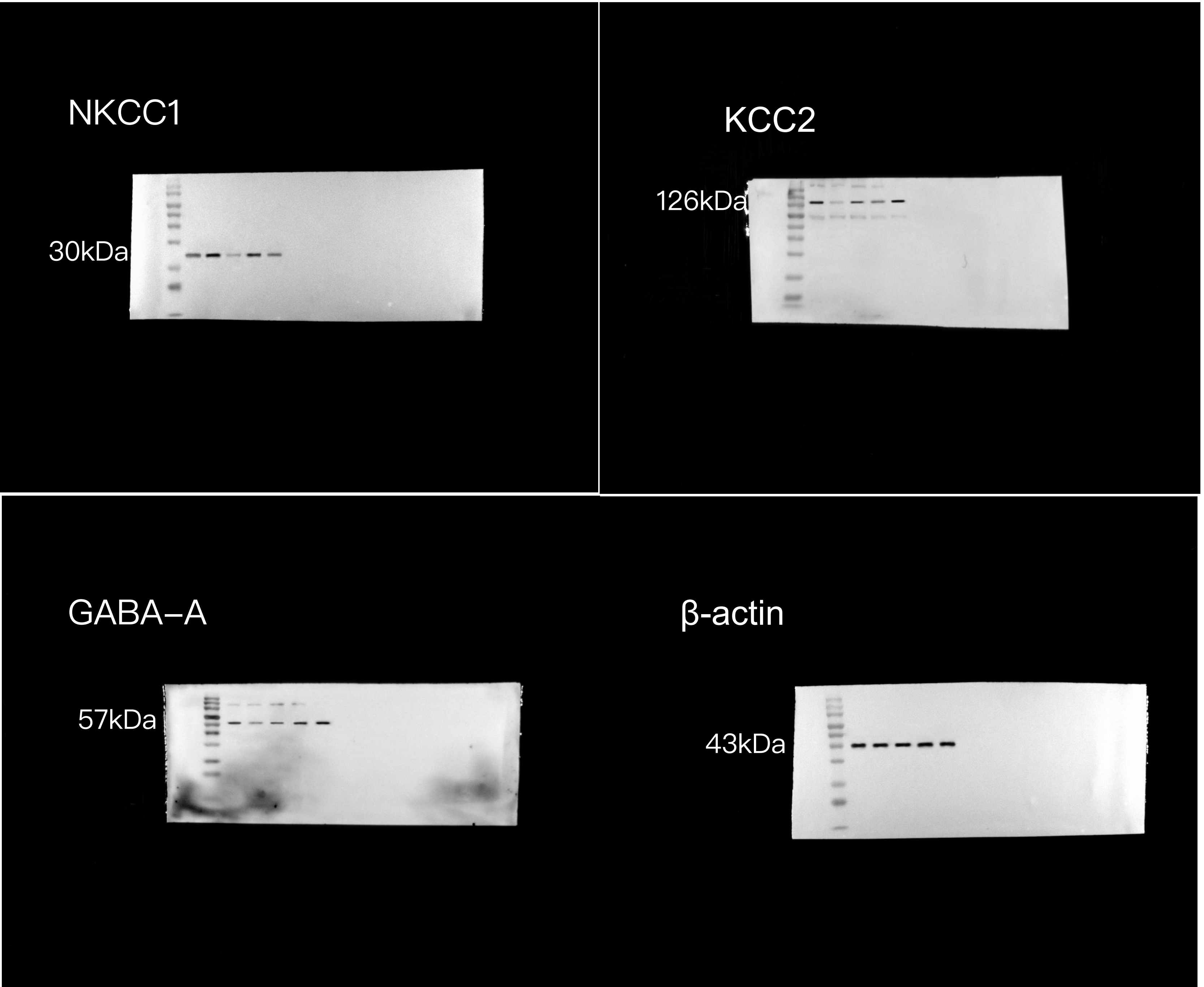

Supplement: Supplementary file 1 [file DataSheet1.pdf]
