# Supplementary figures and images for: Combined use of Bumetanide and MGE cell transplantation alleviates neuropathic pain and its mechanism after spinal cord injury in mice
Source: Front Immunol. 2026 Mar 18;17:1751436. doi: 10.3389/fimmu.2026.1751436 (PMC13038438; doi:10.3389/fimmu.2026.1751436)

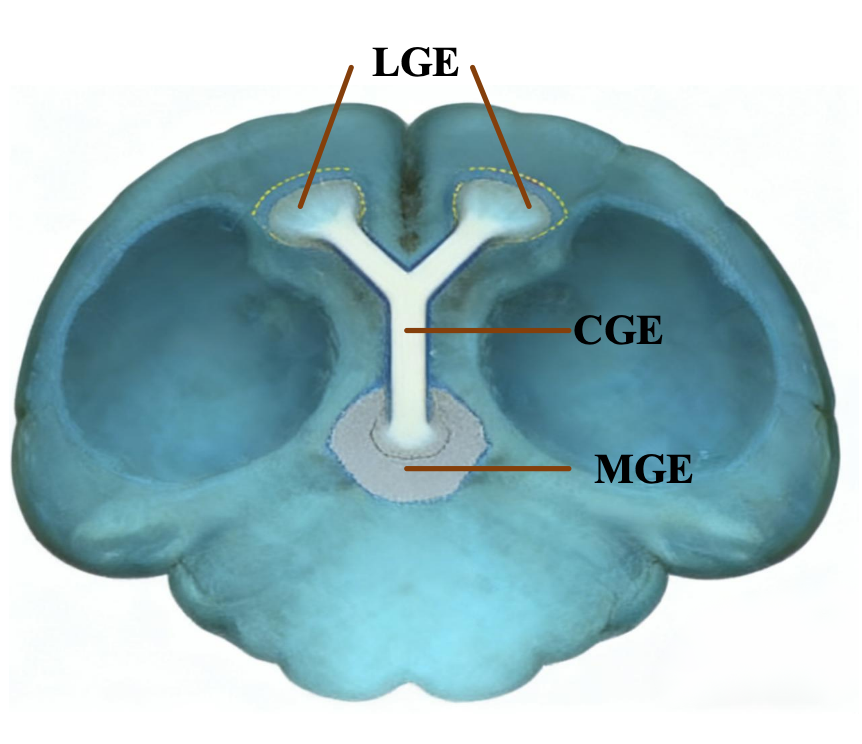

Supplement: Supplementary file 2 [file Image1.tif]
